# Editorial *Materials*: Special Issue on Advances in Luminescent Engineered Nanomaterials

**DOI:** 10.3390/ma14113121

**Published:** 2021-06-07

**Authors:** Luís Pinto da Silva

**Affiliations:** 1 Chemistry Research Unit (CIQUP), Faculty of Sciences of University of Porto, R. Campo Alegre 687, 4169-007 Porto, Portugal; luis.silva@fc.up.pt; 2 LACOMEPHI, GreenUPorto, Department of Geosciences, Environment and Territorial Planning, Faculty of Sciences of University of Porto, R. Campo Alegre 687, 4169-007 Porto, Portugal

Engineered nanomaterials are purposely manufactured particles with sizes typically between 1 and 100 nm, which can be either organic, inorganic, or organometallic in nature. Given their nanoscale, engineered nanomaterials possess properties that are different than bulk materials with the same composition, allowing for new physical and chemical properties. In fact, engineered nanomaterials have been attracting significant attention due to their improved performance, such as emission of light via either down-conversion or up-conversion luminescent pathways, when excited by UV, visible or infrared light.

This Special Issue presents key findings and advances regarding different types of luminescent engineered nanomaterials, with contributions ranging between providing insights into fabrication strategies and development/optimization of practical applications for these materials. The diversity of contributions in this Special Issue highlights the broad appeal related to the topic of luminescent engineered nanomaterials, and their growing importance for the research community. More specifically, here are featured four original research papers as well as two review papers, authored and reviewed by experts in their given fields. Herein, this Special Issue provides valuable contributions for the growing efforts towards research and development of novel luminescent engineered nanomaterials.

Huang et al. [1] employed a novel core-shell and inorganic-organic hybridization strategy to fabricate Eu^3+^-doped UVO_4_ nanoparticles to broaden their excitation spectral bandwidth to the range of 230-415 nm, thereby covering the entire ultraviolet spectrum of solar light. This feature is useful, given that for optoelectronic applications it is desirable for lanthanide-doped phosphors to have a broad excitation spectrum. In fact, while lanthanide-doped YVO_4_ (a high quantum efficient lasing material) can be used in silicon solar cells and convert ultraviolet light to visible light for more efficient power generation, its spectral range is not broad enough to cover the entire ultraviolet spectrum of solar light. Herein, Huang et al. [1] solved this issue by broadening the spectral bandwidth of these materials, enabling further applications in silicon solar cells.

Advanced oxidation processes (AOPs) are proven to be effective for the treatment of wastewater, with heterogeneous photocatalysis being selected as one of the best options for the destruction of many recalcitrant organic pollutants. Currently, pure or modified titanium-dioxide (TiO_2_) nanoparticles are widely used photocatalysts due to the optical and electronic properties of Ti, and their low cost, abundance, chemical stability and non-toxicity. However, the band gap (E_g_ of ~3.20 eV) only allows TiO_2_ to absorb ultraviolet light, inhibiting its industrial applications under visible light. To overcome this problem, El Mragui et al. [2] synthesized TiO_2_ nanoparticles, doped with either Fe or Co, by wet chemical methods (sol-gel and precipitation), and applied them as photocatalysts for carbamazepine under both ultraviolet and visible light irradiation. The addition of both Fe and Co lead to a decrease in charge recombination, while the addition of Fe enhanced the photocatalytic degradation of carbamazepine under both ultraviolet and visible light. Thus, these authors showed that the addition of doping cations can suppress the electron/hole recombination of TiO_2_, enhancing their visible light-induced photocatalytic activity [2].

While the work of El Mragui et al. [2] showed that monodoping TiO_2_ nanoparticles with transition metals is a promising strategy for enhancing their photocatalytic activity, it was not clear if such improvements are enough to offset the potential environmental impacts of adding metal ions to the synthesis of TiO_2_. Fernandes et al. [3] aimed to elucidate this topic by performing a Life Cycle Assessment (LCA) study to determine the sustainability of monodoping TiO_2_ with different transition metals (Fe, Co, Mn and Ni), toward the enhancement of their photocatalytic activity for the degradation of different chemicals under ultraviolet and visible light. The obtained results showed that the addition of the transition metals has no significant effect on the environmental impacts associated with the synthesis of TiO_2_ [3]. Thus, metal-monodoping is shown to be a promising and sustainable strategy for enhancing the photocatalytic performance of TiO_2_ nanoparticles [2,3].

Although nitrogen-doping is a commonly employed strategy for enhancing the fluorescence quantum yield of carbon dots, luminescent carbon-based nanoparticles, there is in existent literature limited information regarding the efficiency and associated environmental impacts of different nitrogen-doping strategies. Herein, Christé et al. [4] prepared carbon dots from citric acid via two different synthesis routes: microwave-assisted hydrothermal treatment with nitrogen-doping by addition of small organic molecules (either urea or ethylenediamine); microwave-assisted solvothermal treatment in nitrogen-containing organic solvents (*n*,*n*-dimethylformamide, acetonitrile or pyridine). The presented results showed that carbon dots synthesized via both hydrothermal routes presented better doping efficiencies (~15 at.%) than all three solvothermal-based strategies (0.6-7 at.%). However, from the former, only the route involving ethylenediamine provided a non-negligible synthesis yield. Furthermore, a life cycle assessment (LCA) study also showed the addition of ethylenediamine as the most sustainable route among all five routes. Thus, this study showed that this route should be the preferred strategy in subsequent studies aiming for the development of nitrogen-doped carbon dots [4].

One of the main current health concerns is antibiotic resistance development, which is outpacing the discovery of novel antibiotics. Thus, researchers are focusing on novel alternatives, such as antimicrobial photodynamic therapy (APDT), to combat pathogens even before infection occurs. Small molecules photosensitizers haven been used in APDT, in which they can destroy pathogens by light-induced generation of reactive oxygen (ROS) species. However, they are frequently limited in widespread application by synthetic expense and complexity. Here, Knoblauch and Geddes [5] performed a review of the state-of-the-art of using carbon dots in APDT, highlighting selected studies, strategies for performance optimization, and use in hybrid disinfection systems and materials. Limitations, challenges, and future perspective are also discussed. Thus, this review provides a relevant foundation for the use of carbon dots as promising novel photosensitizers in APDT [5].

Finally, Aziz et al. [6] performed a comprehensive review on optical properties of polymer electrolytes and composites. In this work, the authors started by discussing the fundamentals, principles, and advantages of different types of polymer electrolytes. Subsequently, the authors highlighted the characteristics and performance of several polymer hosts. Focus was also given to new developments in the investigation of the optical properties of polymer electrolytes. Namely, to the optical bang gap study using the Tauc’s model and optical dielectric loss parameter. In this review paper, the authors were able to demonstrate that both the Tauc’s model and optical dielectric loss should be studied to specify the type of electron transition and estimate the optical band gap accurately.

In conclusion, the studies presented in this Special Issue compile relevant progress in the development of new luminescent engineered nanomaterials. I would like of express my sincere thanks to all authors for their valuable contributions and the reviewers for assisting with their expertise and time, as well as the editorial team of *Materials* for their kind support, without which this Special Issue would not have been possible.
